# Design, Synthesis and Biological Evaluation of 1,3-Diphenyl-3-(phenylthio)propan-1-ones as New Cytotoxic Agents 

**DOI:** 10.22037/ijpr.2021.114865.15076

**Published:** 2021

**Authors:** Maryam Bayanati, Soraya Shahhosseini, Farshad. H. Shirazi, Golrokh Farnam, Afshin Zarghi

**Affiliations:** a *Department of Pharmaceutical Chemistry, School of Pharmacy, Shahid Beheshti University of Medical Sciences, Tehran, Iran. *; b *Protein Technology Research Center, Shahid Beheshti University of Medical Sciences, Tehran, Iran. *; c *Department of Toxicology and Pharmacology, School of Pharmacy, Shahid Beheshti University of Medical Sciences, Tehran, Iran.*

**Keywords:** Synthesis, 1, 3-Diphenyl-3-(phenylthio)propan-1-one, Docking study, Cytotoxic effect, MTT, MCF-7

## Abstract

Cancers in terms of morbidity and mortality are one of the major universal issues. New compounds of anticancer agents based on β-aryl-β-mercapto ketones scaffold possessing piperidinylethoxy or morpholinylethoxy groups were synthesized and evaluated as cytotoxic agents. Cytotoxic effects of synthesized compounds were measured against MCF-7, human ER-positive breast cancer cell lines, using MTT assay. The results indicated that all compounds had high cytotoxic activity on MCF-7 cancerous cells, even more than the reference drug Tamoxifen. Among them, compounds 3-(4-(2-morpholinoethoxy)phenyl)-1-phenyl-3-(phenylthio)propan-1-one (**4a)** and 1-(4-methoxyphenyl)-3-(3-(2-morpholinoethoxy)phenyl)-3-(phenylthio)propan-1-one (**4h)** had no significant cytotoxic effects on normal cells compared to Tamoxifen. Our results also indicated that adding tertiary amine basic side chain, found in Tamoxifen drug, to 1,3-diphenyl-3-(phenylthio)propan-1-ones improves the cytotoxic effects of these compounds on breast cancer cells.

## Introduction

**Figure 1:**
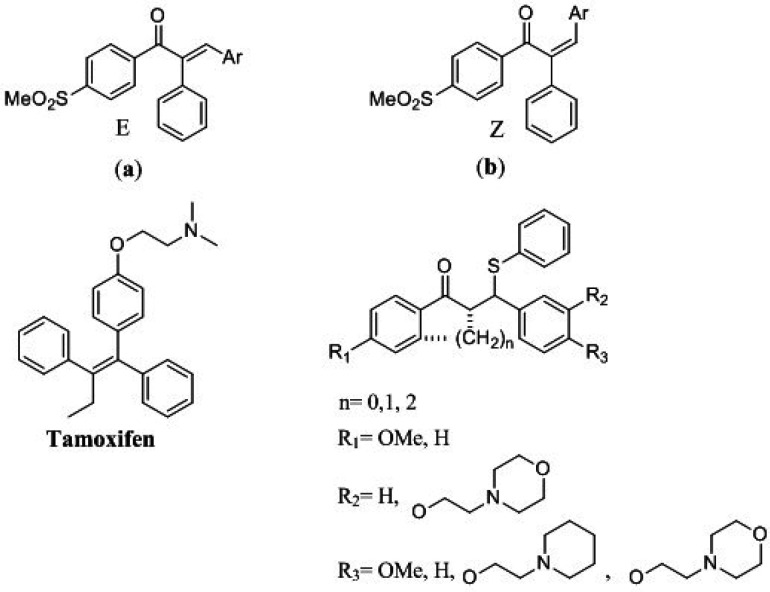
(a and b) Chalcones with cytotoxic effects and Tamoxifen as a lead compound and designed compounds

Cancers in terms of morbidity and mortality are one of the major universal issues. The mortality of cancer is around one in six people, which is the second major cause of death (1). Many structural motifs have been studied for anticancer effects on cancer cells (2-6). Among them, 1, 3-diaryl-2-propen-1-ones have been thoroughly studied for their promising biological activities on this purpose (7-10). 1, 3-diaryl-2-propen-1-ones called chalcones demonstrated various biological activities including anticancer (11-14), anti-Alzheimer (15), antioxidant (16, 17) and antiproliferative (18-20) activities. So for the investigation of anticancer drugs, chalcones are very favorable structures to be explored. Structural adjustment leads to an increase in therapeutic efficacy and biological activity and also a decrease in side effects (21). Recently we reported new classes of potent acyclic chalcone compounds (Figure 1) with cytotoxicity effects (22-25).

Breast cancer has known as the most prevalent cancer in women among all types of cancers. On average, about one in eight women’s lifetimes is diagnosed with breast cancer (26, 27). In this regard, the discovery and introduction of compounds used to treat breast cancer like Tamoxifen are essential. Tamoxifen is renowned for the non-steroidal selective estrogen receptor modulators (SERMs) family that are utilized extensively in early and progressed breast cancer clinical administration. Tamoxifen and many other SERMs, effectively could induce apoptosis and reduce the proliferation rate of cancer cells. SERMs have a flexible tertiary amine side chain that is thought to be responsible for their anticancer effects (28-30). 

In our recent work, we have introduced a new ferrocene chalcone-based scaffold as SERMs which, apart from chalcone scaffold, also has the aminoethoxy typical side chain of SERMs. Our biological results exhibited that these compounds have cytotoxic effects with the ability to induce apoptosis (31). To find cytotoxic agents and consider chalcones as biologically active compounds against cancer cells and Tamoxifen as an anti-breast cancer compound, a new group of 1,3-diphenyl-3-(phenylthio)propan-1-ones were designed, synthesized, and evaluated, which possess tertiary amine moiety as typical pharmacophore of SERMs. The cytotoxic activities of these β-aryl-β-mercapto ketones compounds were evaluated against MCF-7 to investigate their cytotoxic effects.

## Experimental

*General*

All chemicals, reagents, and solvents were obtained from Aldrich Chemical Co., Acros Co., and Merck AG Chemical Co. and used without more purification. Melting points were recorded with Thomas–Hoover capillary instrument. Infrared spectra were recorded on Cary 630 FTIR spectrometer using KBr pellets for solid samples and reported in ʋ_max_ (cm^-1^) scale. NMR spectra were recorded with a Varian - INOVA 500MHz instrument using CDCl_3_ as a solvent and trimethylsilane (TMS) as a standard internal reference. Chemical shifts are reported in ppm (δ) scale. The mass spectral measurements were obtained by a 6410 Agilent LC-MS triple quadrupole mass spectrometer (LC-MS) with an electrospray ionization (ESI) interface.

*Chemistry*

*General procedure for the synthesis of chalcones* ***(3)***

**Scheme 1:**
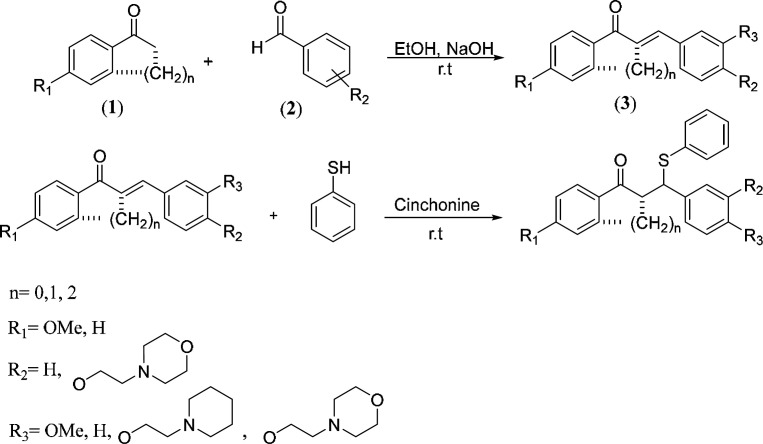
Synthesis of series substituted 1,3-diarylpropane-1-one

At first, a sodium hydroxide*, *10% (w/v) aqueous solution was added (3 mL) to under stirring solution of ketone **1** (1 mmol) and substituted arylaldehyde containing aminoethoxy side chain **2 **(1 mmol) in ethanol; and upon completion of the substrates (TLC), if the product was solid (**3a-g**) filtered, and washed with cold ethanol and dried to obtain the pure chalcone (Scheme 1). However, if the product was oily (**3h-i**), at first, the solvent was evaporated under vacuum, and then the product was extracted with ethyl acetate and water (1:1) (24).

*General procedure for synthesis of* *((N, N-dialkylaminoethoxy)phenyl)-1-phenyl-3-(phenylthio)propan-1-one derivatives (****4a-i****)*

At first, 2.0 mmol of substituted chalcone was added to a stirred mixture of cinchonine (8.8 mg, 1.5% mol) in chloroform (4 mL), then 3 mmol of thiol was added to under stirring above mixture (32, 33). Stirring was continued until TLC showed completion of the reaction. Then, the solvents were evaporated in a vacuum. For solid products (**4a-g**) the residue was washed with hexane and crystallized in ethyl acetate. But for oily products (**4h-i**), the residue was first dissolved in methanol and then extracted with n-hexane, and the products were further purified by column chromatography using a chloroform:methanol solvent system (85%:15%).

*3-(4-(2-morpholinoethoxy)phenyl)-1-phenyl-3-(phenylthio)propan-1-one* ***(4a)***

Yield, 89%; white powder; mp: 93-95 °C; IR (KBr): ν (cm^−1^) (1677 CO); ^1^H NMR (CDCl_3_): 2.56 (t, 4H, morpholine -NCH_2_), 2.77 (t, 2H, -NCH_2_), 3.54 (d, 1H, CH_2 _*J *= 8 HZ), 3.61 (d, 1H, CH_2_* J *= 8 HZ), 3.72 (t, 4H, morpholine -OCH_2_), 4.05 (t, 2H, -OCH_2_), 4.93 (dd, 1H, CH), 6.78 (d, 2H, phenoxy H_3_ & H_5 _*J *= 8 HZ), 7.20-7.34 (m, 7H, phenoxy H_2_ & H_6_ & phenylthio); 7.40-7.43 (m, 2H, phenyl H_3_ & H_5_); 7.51-7.55 (m, 1H, phenyl H_4_); 7.86 (d, 2H, phenyl H_2_ & H_6 _*J *= 8 HZ); ^13^C NMR (CDCl_3_): 44.82, 47.60, 54.08, 57.63, 65.67, 66.89, 114.51, 127.48, 128.08, 128.63, 128.89, 128.91, 132.65, 133.28, 133.33, 134.42, 136.72, 157.87, 197.15; LC-MS (ESI) m/z: 448 (M+1).

*1-phenyl-3-(phenylthio)-3-(4-(2-(piperidin-1-yl)ethoxy)phenyl)propan-1-one* ***(4b)***

Yield, 86%; white powder; mp: 94-96 °C; IR (KBr): ν (cm^−1^) (1681 CO); ^1^H NMR (CDCl_3_): 1.86 (bs, 4H, piperidine H_3 _& H_5_), 2.24 (bs, 2H, piperidine H_4_), 2.80 (bs, 2H, -NCH_2_), 3.36 (bs, 2H, CH_2_), 3.51-3.64 (m, 4H, piperidine H_2 _& H_6_), 4.49 (d, 2H, -OCH_2_ *J *= 8 HZ), 4.92 (q, 1H, -CH *J *= 5.5 HZ & *J *= 5.5 HZ), 6.77 (d, 2H, phenoxy H_3_ & H_5_ *J *= 9 HZ), 7.23-7.53 (m, 10H, phenoxy H_2_ & H_6 _& phenylthio & phenyl H_3_ & H_4_ & H_5_), 7.55 (d, 2H, phenyl H_2 _& H_6_*J* = 7 HZ); ^13^C NMR (CDCl_3_): 21.86, 22.72, 44.76, 47.53, 53.86, 56.19, 62.68, 114.45, 127.57, 128.03, 128.64, 128.92, 129.15, 132.67, 133.31, 134.17, 134.62, 136.64, 156.30, 197.00; LC-MS (ESI) m/z: 446 (M+1).

*1-(4-methoxyphenyl)-3-(4-(2-morpholinoethoxy)phenyl)-3-(phenylthio)propan-1-one* ***(4c)***

Yield, 86%; white powder; mp: 106-108 °C; IR (KBr): ν (cm^−1^) (1666 CO); ^1^H NMR (CDCl_3_): 2.58 (s, 4H, morpholine -NCH_2_), 2.78 (t, 2H, -NCH_2_), 3.48-3.52 (m, 2H, CH_2_), 3.74 (t, 4H, morpholine -OCH_2_), 3.87 (s, 3H, OMe), 4.07 (t, 2H, -OCH_2_), 4.95 (t, 1H, CH), 6.80 (d, 2H, phenoxy H_3_ & H_5 _*J *= 8.5 HZ), 6.91 (d, 2H, 4-methoxyphenyl H_3_ & H_5_ *J *= 8.5 HZ); 7.24-7.36 (m, 7H, phenoxy H_2_ & H_6_ & phenylthio); 7.87 (d, 2H, 4-methoxyphenyl H_2_ & H_6_* J *= 8.5 HZ); ^13^C NMR (CDCl_3_): 24.21, 25.96, 44.04, 47.79, 55.04, 55.47, 57.93, 65.91, 113.73, 114.48, 114.99, 127.34, 128.81, 128.83, 130.37, 132.58, 133.24, 134.55, 157.99, 163.57, 195.63; LC-MS (ESI) m/z: 478 (M+1).

*1-(4-methoxyphenyl)-3-(phenylthio)-3-(4-(2-(piperidin-1-yl)ethoxy)phenyl)propan-1-one* ***(4d)***

Yield, 91%; white powder; mp: 107-108 °C; IR (KBr): ν (cm^−1^) (1666 CO); ^1^H NMR (CDCl_3_): 1.45 (d, 2H, piperidine H_4 _*J *= 4 HZ), 1.61 (t, 4H, piperidine H_3 _& H_5_), 2.49 (s, 4H, piperidine H_2 _& H_6_), 2.74 (s, 2H, -NCH_2_), 3.47-3.60 (m, 2H, CH_2_), 3.86 (s, 3H, OMe), 4.05 (t, 2H, -OCH_2_), 4.94 (t, 1H, CH), 6.79 (d, 2H, phenoxy H_3_ & H_5_ *J *= 8 HZ), 6.90 (d, 2H, 4-methoxyphenyl H_3_ & H_5 _*J *= 8.5 HZ), 7.23-7.35 (m, 7H, phenoxy H_2_ & H_6 _& phenylthio), 6.87 (d, 2H, 4-methoxyphenyl H_2_ & H_6 _*J *= 8.5 HZ); ^13^C NMR (CDCl_3_): 24.21, 25.96, 44.40, 47.79, 55.04, 55.47, 57.93, 65.91, 113.73, 114.48, 114.99, 127.34, 128.81, 128.83, 130.37, 132.58, 133.24, 134.55, 157.99, 163.57, 195.63; LC-MS (ESI) m/z: 476 (M+1).

*2-((phenylthio)(4-(2-(piperidin-1-yl)ethoxy)phenyl)methyl)-2,3-dihydro-1H-inden-1-one* ***(4e)***

Yield, 56%; white powder; mp: 95-97 °C; IR (KBr): ν (cm^−1^) (1692 CO); ^1^H NMR (CDCl_3_): 1.62 (bs, 2H, piperidine H_4_), 2.03 (bs, 4H, piperidine H_3 _& H_5_), 3.19 (bs, 4H, piperidine H_2 _& H_6_), 3.40 (bs, 4H, N-CH_2 _& CH_2_), 3.99 (bs, 2H, O-C*H*_2_), 4.59 (bs, 2H, CH & SCH), 6.98 (d, 2H, phenoxy H_3_ & H_5_ *J* = 8 HZ), 7.27-7.31 (m, 2H, phenoxy H_2_ & H_6_),7.40-7.63 (m, 8H, phenylthio & 1-indanone H_3_ & H_4_ & H_5_ ), 7.89 (d, 1H, 1-indanone H_2 _*J *= 8 HZ); ^13^C NMR (CDCl_3_): 22.06, 23.03, 32.43, 54.17, 56.34, 63.23, 115.05, 124.32, 126.18, 127.17, 127.47, 127.66, 129.19, 139.53, 130.69, 132.61, 133.10, 133.32, 134.52, 138.08, 149.50, 158.43, 194.37; LC-MS (ESI) m/z: 458 (M+1).

*2-((4-(2-morpholinoethoxy)phenyl)(phenylthio)methyl)-3,4-dihydronaphthalen-1(2H)-one* ***(4f)***

Yield, 42%; white powder; mp: 97-99 °C; IR (KBr): ν (cm^−1^) (1666 CO); ^1^H NMR (CDCl_3_): 1.80 (bs, 2H, CH_2_), 2.58 (bs, 4H, morpholine -NCH_2_), 2.78 (t, 2H, N-CH_2_), 2.86 (t, 2H, CH_2_-CH_2_), 3.77 (t, 4H, morpholine O-C*H*_2_), 4.06 (t, 2H, O-C*H*_2_), 4.18 (t, 2H, CH & SCH), 7.13-7.52 (m, 13H, Ar); ^13^C NMR (CDCl_3_): 24.65, 27.24, 50.17, 52.44, 54.09, 55.02, 57.60, 66.89, 113.99, 114.56, 126.89, 127.52, 128.16, 128.60, 128.91, 129.48, 130.51, 131.28, 131.75, 136.58, 143.05, 159.05, 197.04; LC-MS (ESI) m/z: 474 (M+1).

*2-((phenylthio)(4-(2-(piperidin-1-yl)ethoxy)phenyl)methyl)-3,4-dihydronaphthalen-1(2H)-one* ***(4g)***

Yield, 40%; white powder; mp: 95-97 °C; IR (KBr): ν (cm^−1^) (1662 CO); ^1^H NMR (CDCl_3_): 1.28 (bs, 6H, piperidine *H*_3_ & *H*_4_ & *H*_5_), 1.78 (bs, 2H, C*H*_2_), 2.56 (bs, 4H, piperidine *H*_2_ & *H*_6_), 2.85 (t, 4H, CH_2_-C*H*_2_ & N-C*H*_2_), 3.76 (t, 2H, O-C*H*_2_), 4.18 (t, 2H, CH & SCH), 7.14-7.50 (m, 13H, Ar); ^13^C NMR (CDCl_3_): 27.25, 28.81, 52.46, 54.11, 55.03, 57.56, 57.69, 65.90, 66.93, 113.99, 114.36, 114.56, 126.55, 126.98, 128.07, 128.59, 128.68, 129.47, 130.97, 131.74, 133.11, 136.58, 157.67, 195.51; LC-MS (ESI) m/z: 472 (M+1).

*1-(4-methoxyphenyl)-3-(3-(2-morpholinoethoxy)phenyl)-3-(phenylthio)propan-1-one* ***(4h)***

Yield, 38%; oily brown liquid; IR (KBr): ν (cm^−1^) (1666 CO); ^1^H NMR (CDCl_3_): 2.58 (bs, 4H, morpholine -NCH_2_), 2.80 (t, 2H, CH_2_N), 3.45-3.50 (m, 2H, CH_2_), 3.73-3.75 (m, 4H, morpholine -OCH_2_), 3.81 (s, 3H, OMe), 4.02 (t, 1H, CH), 4.11 (t, 2H, OCH_2_), 6.78-6.86 (m, 3H, phenoxy H_2_ & H_4_ & H_6_), 7.45-7.58 (m, 6H, phenoxy H_5_ & phenylthio H_3 _& H_4 _& H_5 _& 4-methoxyphenyl H_3_ & H_5_); 7.96 (d, 4H, 4-methoxyphenyl H_2_ & H_6_& phenylthio H_2 _& H_6 _*J *= 7.5 HZ); ^13^C NMR (CDCl_3_): 36.89, 45.09, 54.08, 55.93, 57.51, 66.89, 66.96, 111.97, 113.73, 119.89, 122.05, 123.22, 124.53, 128.13, 128.59, 133.08, 136.39, 136.95, 148.02, 148.32, 158.34, 198.66; LC-MS (ESI) m/z: 478 (M+1).

*3-(4-methoxy-3-(2-morpholinoethoxy)phenyl)-1-(4-methoxyphenyl)-3- (phenylthio)propan-1-one* ***(4i)***

Yield, 38%; oily brown liquid; IR (KBr): ν (cm^−1^) (1666 CO); ^1^H NMR (CDCl_3_): 2.60 (bs, 4H, morpholine -NCH_2_), 2.65 (bs, 2H, -NCH_2_), 3.46-3.61 (m, 2H, CH_2_), 3.75-3.78 (m, 4H, morpholine -OCH_2_), 3.81 (s, 3H, OMe), 3.86 (s, 3H, OMe), 4.06-4.15 (m, 2H, -OCH_2_), 4.24 (t, 1H, CH), 6.88-6.92 (m, 3H, phenoxy H_2_ & H_5_ & H_6_), 7.21-7.34 (m, 9H, phenylthio & 4-methoxyphenyl); ^13^C NMR (CDCl_3_): 29.70, 44.37, 48.14, 54.07, 55.48, 55.88, 57.45, 66.93, 111.51, 112.62, 112.62, 113.75, 119.87, 120.57, 123.50, 128.83, 130.37, 130.71, 132.63, 133.73, 143.97, 147.90. 195.54; LC-MS (ESI) m/z: 508 (M+1).

*Cytotoxicity* *evaluation using MTT assay method*

To evaluate the cytotoxic effects of synthesized compounds against MCF-7, human cancerous cell lines, the MTT (3-(4, 5-dimethylthiazol-2-yl)-2, 5-diphenyltetrazolium bromide)) the assay was performed.

For evaluation of the antiproliferative activity of designed and synthesized compounds, a human tumor cell line, MCF-7, was procurement from the Iranian Biological Resource Center (IBRC), Tehran, Iran(19-21). In the RPMI1640 medium, the cells were cultured under a high humidity atmosphere with 5% CO_2_ at 37 °C. Then, the cells were enriched with 10% fetal bovine serum (FBS) plus 100 µg/mL streptomycin. Using the MTT method, cell viability was evaluated, relying on the reduction of MTT dye by mitochondrial succinate dehydrogenase enzyme to produce formazan crystals in purple color in living cells. The cells in 96-well plate format were prepared at a concentration of 10^4^ cells/well and, for 24 h allowed to incubate. With 1 and 100 μM of synthesized compounds in water/DMSO, the cells were incubated at 37 °C for 48 h. Tamoxifen treated cells as the positive control and intact cells as the negative group was used. After incubation, 10 μL of MTT was added, and the cells were incubated at 37 °C for 4hr. The supernatant was removed, and cells were exposed with 100 μL DMSO for 20 min at 37 °C. The absorbance of cells (OD) at 570 nm by a spectrophotometer plate reader (Infinite® M200, TECAN) was determined, and the percentage of inhibition was calculated. 

*Molecular modeling studies*

 Docking studies were implemented by AutoDock 4 software. The X-ray crystallography of lasofoxifene as a selective estrogen receptor modulator bound to the human estrogen receptor α was retrieved from the protein data bank server [PDB code: 2OUZ]. The crystal structure was optimized by removing all water molecules, polar hydrogens were added to the protein, and then Kollman united partial atomic charges were appropriate. At last, the format of the prepared file was converted to PDBQT format using AutoDock 4. The energy of prepared ligand molecules was minimized through the MM+ method with HyperChem 8.0 software; the PDBQT format of the ligands was obtained using AutoDock tools. A grid box of 20-20-20 Å with the x, y, and z directions around the active site of the protein was constructed. The Lamarckian genetic search algorithm was utilized with a total of 50 runs. For efficiency, from the docking box, protein residues with atoms greater than 7.5 Å were removed. At the end of the process, an optimum conformation with the relative lower binding energy was selected for each compound.

## Results and Discussion

The cytotoxic effect of synthesized compounds on the viability of cancerous cells *in-vitro*, ER-α-positive MCF7 was evaluated using MTT assay. The antiproliferative activities of the synthesized compounds were compared with the reference drug, Tamoxifen at the concentration of 1 μM on MCF-7 cells and 100 μM on fibroblast cells, respectively.

The MTT test results of the synthesized compounds have been shown in Table 1. All of the synthesized compounds showed better cytotoxic effects on the MCF7 cell line compared with Tamoxifen as a reference drug. However, there was no significant difference between the cytotoxic effects of the synthesized compounds.In addition, the results of normal cells (fibroblasts) indicated that the synthesized compounds with the concentration of 100X against cancerous cells showed less cytotoxic effects than Tamoxifen, and compounds **4a** and especially **4h** compared with Tamoxifen displayed little cytotoxic activity on these cells. According to the structural similarity of these two compounds, it can be concluded that the linear structures with polar morpholine group as a polar side chain showed decreasing cytotoxicity on the normal cell in comparison with piperidinyl as a hydrophobic side chain. According to cytotoxic results, the synthesized compounds demonstrated acceptable effects on the MCF-7 cell line. 

**Figure 2:**
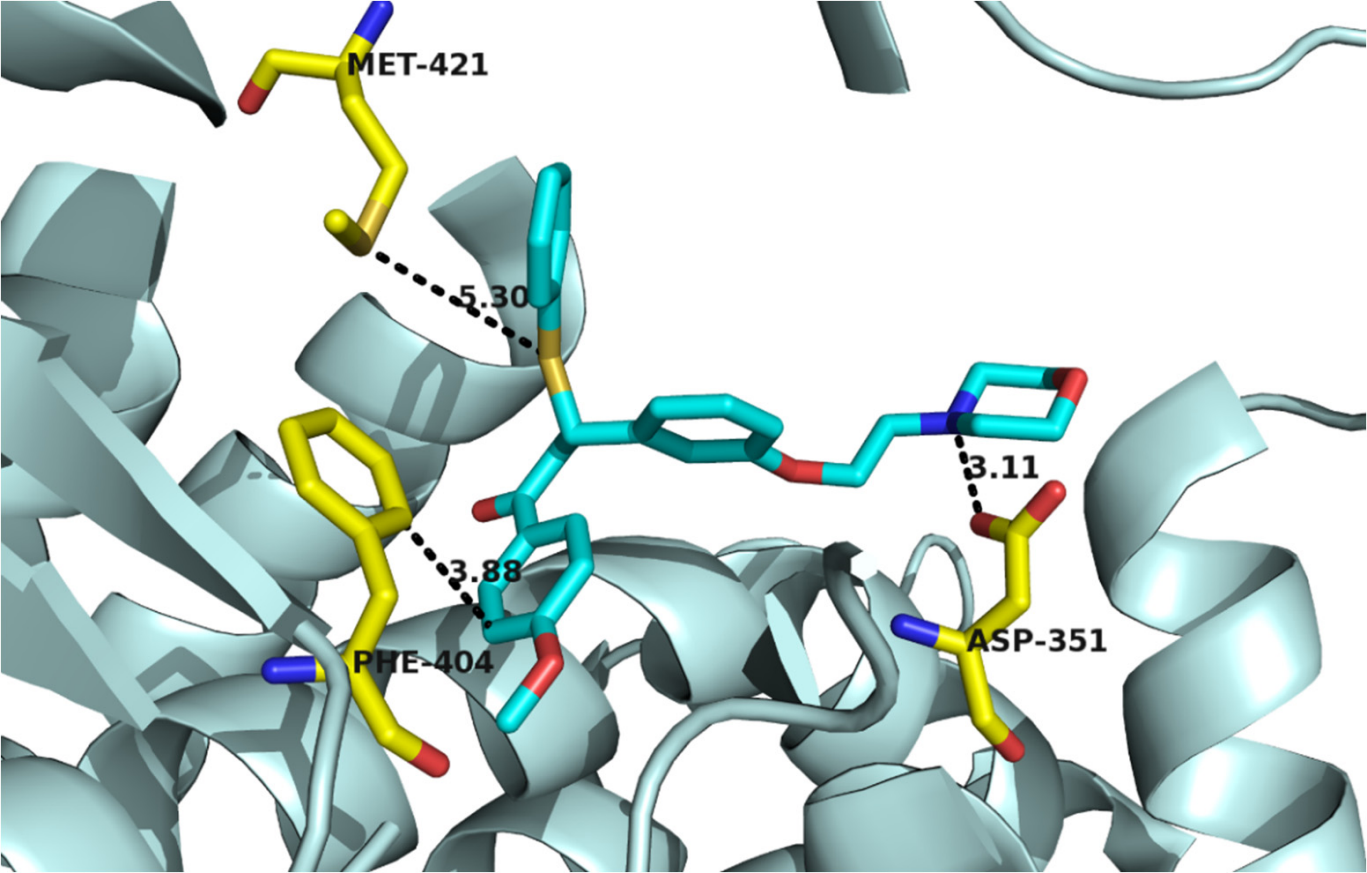
Important interactions measure of **4h **docked in the active site of ERα

Considering the MTT evaluation results and structural correspondence between designed ((N, N-dialkylaminoethoxy)phenyl)-1-phenyl-3-(phenylthio)propan-1-one derivatives (**4a-i**) and Tamoxifen, one of the mechanisms proposed to explain the cytotoxicity effects of these compounds on MCF-7 cancerous cells, is estrogen receptors blockade. Based upon, to display the interactions with estrogen receptor α (ERα), compound **4h**, one of the most potent compounds with less cytotoxic effects on normal cells, was docked in ERα active site (Figure 2). The molecular modeling study demonstrated that the compound **4h** was well bound into the active site of receptor estrogen receptor alpha. The tertiary amine basic side chain (diethylaminophenoxy) was well placed into the active site of a protein, and the N atom of this side chain formed a hydrogen bond with the carboxylate group of Asp^351 ^(distance = 3.11 Å). Also, a disulfide bond formed between the S atom of compound **4h** and the S atom of Met^421^ (distance = 5.30 Å). Π-Π interaction between phenyl ring of Phe^404^ and C-1 phenyl ring of **4h **(distance = 3.88 Å) was also observed. According to these data, it could realize that one of the probable mechanisms of action of these compounds may be mediated through interference with estrogen receptors. However, binding studies are needed to prove this point.

**Table 1:** Cytotoxicity effects of the synthesized compounds

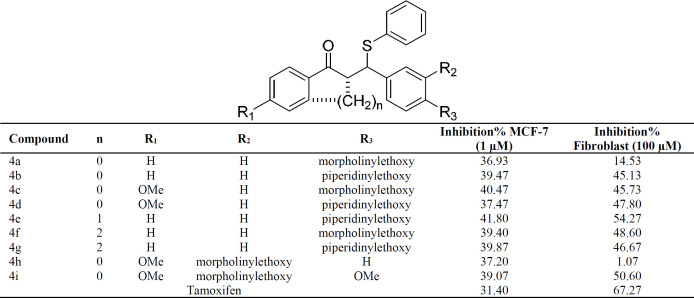

## Conclusion

The results showed that the synthesized compounds had significant cell cytotoxicity on breast cancer cells with a low toxicity effect on normal cells. Our results indicated that adding tertiary amine basic side chain, found in Tamoxifen drug, to 1,3-diphenyl-3-(phenylthio)propan-1-ones improves the cytotoxic effects of these compounds on breast cancer cells. To better understand the mechanism of action of these compounds, they should be tested on other types of cancer cells as well as their binding studies should be implemented.
